# Parallel evolution of the *POQR* prolyl oligo peptidase gene conferring plant quantitative disease resistance

**DOI:** 10.1371/journal.pgen.1007143

**Published:** 2017-12-22

**Authors:** Thomas Badet, Derry Voisin, Malick Mbengue, Marielle Barascud, Justine Sucher, Pierre Sadon, Claudine Balagué, Dominique Roby, Sylvain Raffaele

**Affiliations:** LIPM, Université de Toulouse, INRA, CNRS, Castanet-Tolosan, France; John Innes Centre, UNITED KINGDOM

## Abstract

Plant pathogens with a broad host range are able to infect plant lineages that diverged over 100 million years ago. They exert similar and recurring constraints on the evolution of unrelated plant populations. Plants generally respond with quantitative disease resistance (QDR), a form of immunity relying on complex genetic determinants. In most cases, the molecular determinants of QDR and how they evolve is unknown. Here we identify in *Arabidopsis thaliana* a gene mediating QDR against *Sclerotinia sclerotiorum*, agent of the white mold disease, and provide evidence of its convergent evolution in multiple plant species. Using genome wide association mapping in *A*. *thaliana*, we associated the gene encoding the POQR prolyl-oligopeptidase with QDR against *S*. *sclerotiorum*. Loss of this gene compromised QDR against *S*. *sclerotiorum* but not against a bacterial pathogen. Natural diversity analysis associated *POQR* sequence with QDR. Remarkably, the same amino acid changes occurred after independent duplications of *POQR* in ancestors of multiple plant species, including *A*. *thaliana* and tomato. Genome-scale expression analyses revealed that parallel divergence in gene expression upon *S*. *sclerotiorum* infection is a frequent pattern in genes, such as *POQR*, that duplicated both in *A*. *thaliana* and tomato. Our study identifies a previously uncharacterized gene mediating QDR against *S*. *sclerotiorum*. It shows that some QDR determinants are conserved in distantly related plants and have emerged through the repeated use of similar genetic polymorphisms at different evolutionary time scales.

## Author summary

Plant disease resistance is mediated by molecular components depending on pathogens infection strategy. *Sclerotinia sclerotiorum* is a devastating plant pathogenic fungus notorious for its ability to infect a wide variety of plant species by rapidly triggering cell death. Plant exhibit a response designated as quantitative disease resistance (QDR) when challenged by *S*. *sclerotiorum*, the molecular bases of which are largely unknown. Here we used genome wide association mapping a natural population of *Arabidopsis thaliana* host to identify the POQR prolyl oligopeptidase gene involved in QDR against *S*. *sclerotiorum*, and validate its function. We associate specific amino-acid changes in POQR sequence to increased QDR in *A*. *thaliana* accessions. Remarkably, the same changes occurred in multiple plant lineages. We demonstrate the parallel evolution of *POQR* in *A*. *thaliana* (*Brassicaceae*) and tomato (*Solanaceae*), showing that this gene evolved through duplication followed by similar sequence and expression divergence in these two distant plant lineages. These findings expand on the diversity of molecular functions involved in QDR and suggest that the evolution of QDR against broad host range pathogens is repeatable to some extent.

## Introduction

Plant pathogens are major threats to biodiversity in natural ecosystems and to food security worldwide. Plant disease resistance is mediated by an elaborate multilayered system of defense, sometimes including resistance (*R*) genes conferring complete resistance against a limited number of pathogen genotypes and encoded by nucleotide-binding site leucine-rich repeat (NBS-LRR) proteins [1]. Substantial progress has been made in understanding the genetic and molecular bases of R-gene mediated resistance [2,3]. However, for some important plant diseases, especially those caused by necrotrophic and broad host range pathogens, *R* genes of major effect are unknown. The only available form of resistance to these diseases is Quantitative Disease Resistance (QDR). QDR is based on complex inheritance, involving numerous genes of small effect [4–6]. QDR is frequent in crops and natural plant populations, and is of practical importance in agriculture because it is often more durable than R-mediated resistance [7]. In addition, the identification of genes underlying QDR is expected to provide fundamental insights into the diversity of plant immune responses and prediction of evolutionary trajectories of natural populations. To date, the molecular bases of QDR have been identified only in few cases and involve remarkably diverse molecular functions [6,8,9].

*Sclerotinia sclerotiorum* is an Ascomycete fungus, causal agent of the white mold and stem rot diseases. It is considered as a typical necrotrophic pathogen, using secreted proteins and metabolites to rapidly kill host cells and complete its infection cycle [10,11]. *S*. *sclerotiorum* is also notorious for its remarkably broad host range, encompassing several hundred Eudicot species in about a hundred botanical families [12,13]. *S*. *sclerotiorum* notably infects soybean, tomato and rapeseed on which it causes several hundred million dollar losses annually [14,15]. Besides rapeseed, *S*. *sclerotiorum* is also a natural pathogen of other *Brassicaceae* such as *Arabidopsis* species [16]. On *A*. *thaliana* natural populations, *S*. *sclerotiorum* causes symptoms ranging from high susceptibility to relative tolerance, corresponding to a typical QDR response [17]. The role of a few *A*. *thaliana* genes in resistance to *S*. *sclerotiorum* is beginning to emerge (for a review: [18]), notably through association genetics approaches [19]. Thanks to its reduced linkage disequilibrium and extensive genotyping information, *A*. *thaliana* is an excellent model to deploy genome wide association (GWA) mapping to identify QDR genes [20,21].

A better understanding of plant QDR genes function and molecular evolution is critical to increase the durability of disease resistance mechanisms used in the field, a major challenge for plant breeding and evolutionary biology. *S*. *sclerotiorum* lineage gained the ability to infect *Brassicacea* and *Solanaceaea* plants, among others, after the divergence with *S*. *trifoliorum* lineage, about 8.2 million years ago [13]. The divergence between ancestors of the *Brassicaceae* and *Solanaceaea* plant families is estimated to about 120 million years ago [22]. While most NBS-LRR genes show dramatic lineage-specific expansions and contractions with diverse rates of sequence variation [23,24], a few NBS-LRR gene clusters have likely been conserved over 100 million years in core eudicot genomes [25]. The persistence of function and polymorphism after several million years of divergence has been documented in some orthologous *R* genes such as *Rpm1* in *A*. *thaliana* [26] or members of the *Mla* family in *Triticaea* [27]. This pattern is often indicative of balancing selection acting on resistant and susceptible haplotypes [28]. Similar balancing selection has been identified for the *RKS1* gene conferring quantitative disease resistance to the bacterial pathogen *Xanthomonas campestris* pv. *campestris* (*Xcc*) in *A*. *thaliana* [21]. However, our knowledge of the mechanisms underlying the evolution of QDR genes is very limited. Furthermore, how genes associated with QDR against *S*. *sclerotiorum* evolved in the multiple lineages it infects remains largely unknown.

In this work, we reveal the parallel evolution, in distinct plant lineages, of sequence and expression polymorphisms associated with quantitative disease resistance against the fungal pathogen *S*. *sclerotiorum*. Using genome wide association mapping in *A*. *thaliana* populations, we associated the prolyl-oligopeptidase (POP) gene *POQR* with quantitative disease resistance against *S*. *sclerotiorum*. The phenotypic analysis of two null mutant lines confirmed that *POQR* confers partial resistance to *S*. *sclerotiorum*. Next, we highlight the long term co-existence of *POQR* alleles in *A*. *thaliana* and associate high level of disease resistance with specific amino acid substitutions. Furthermore, similar amino acid substitutions occurred independently in POQR in distinct plant lineages, following independent gene duplications. Genome wide analysis of the POP family in *A*. *thaliana* and *S*. *lycopersicum* indicated that the emergence of POQR resulted from parallel (i) gene duplications, (ii) amino acid substitutions and (iii) gain of gene induction upon *S*. *sclerotiorum* challenge in these two species. We report parallel divergence in gene expression upon *S*. *sclerotiorum* infection for *POQR* and multiple genes that duplicated both in *A*. *thaliana* and *S*. *lycopersicum*. Our findings provide one of the very few functions known for prolyl-oligopeptidases in plants and reveal that the molecular evolution of quantitative resistance against generalist pathogens can follow the same trajectory several times independently in distinct lineages.

## Results

### Genome wide association mapping in a European *A*. *thaliana* population associates *At1g20380* with quantitative disease resistance against *S*. *sclerotiorum*

**Fig 1:**
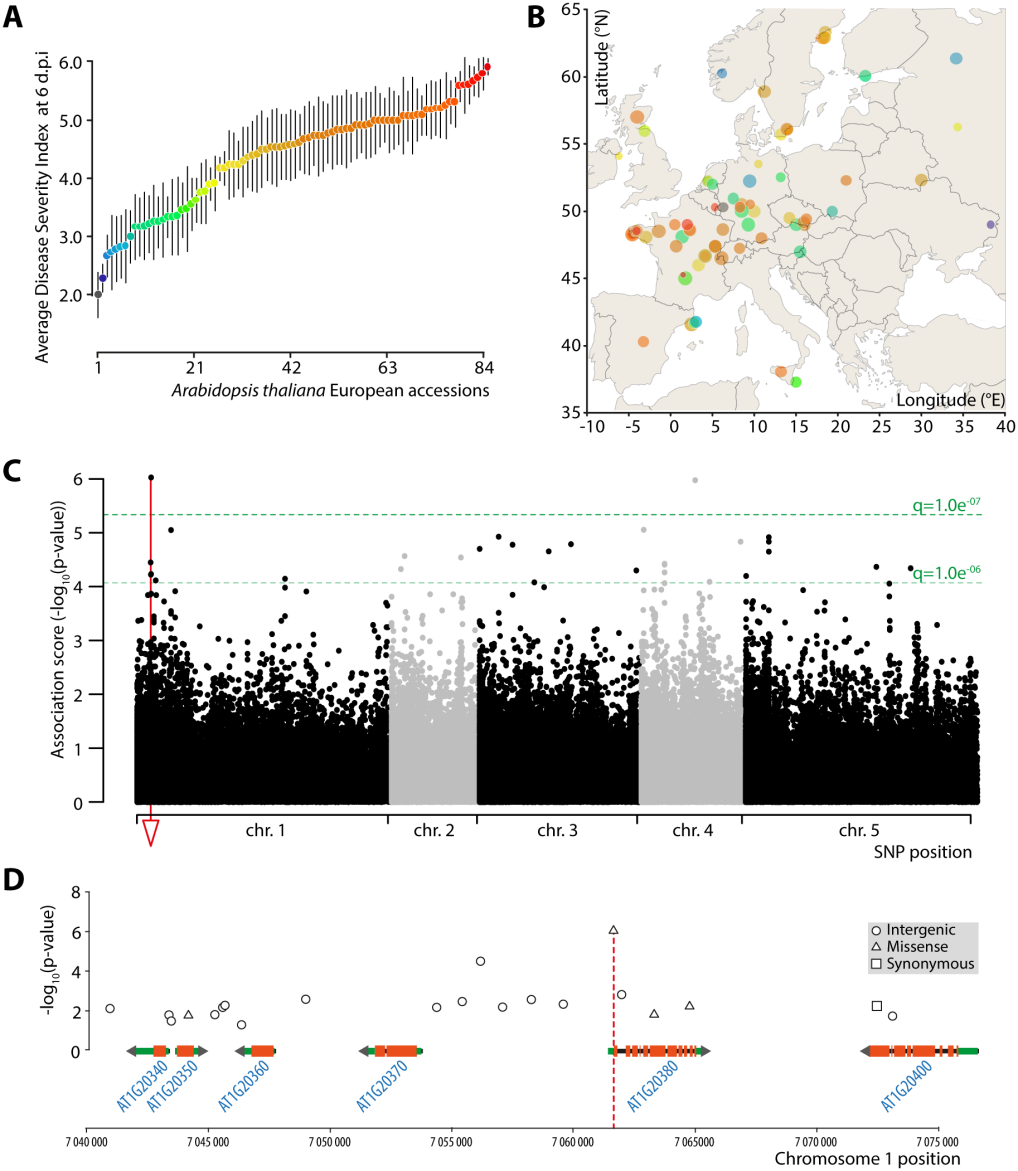
Genome wide association mapping in a European *A*. *thaliana* population associates *At1g20380* with quantitative disease resistance against *S*. *sclerotiorum*. **(A)** Distribution of disease severity index (DSI) at 6 days after inoculation by *S*. *sclerotiorum* in 84 *A*. *thaliana* European accessions. Values shown are averages for 6 to 16 plants per genotype, with error bars showing standard deviation. Points are colored from blue to red according to average DSI. **(B)** Geographic origin of the accessions used in this work, in relation with their average DSI (same color code as in A). Points are sized according to DSI standard deviation. **(C)** Manhattan plot of GWAS results for DSI after S. *sclerotiorum* inoculation. Dotted green lines show false discovery rate thresholds, the red line show the position of the *POQR* locus. Chr., chromosome. **(D)** Close-up of the major association peak centered on *POQR* locus. Only SNPs with association score greater than 1.25 are shown.

To identify genetic loci associated with QDR to *S*. *sclerotiorum*, we used Genome Wide Association (GWA) mapping in *A*. *thaliana* natural populations. For this, we scored disease index six days after leaf inoculation on 84 *A*. *thaliana* European accessions. We used the average disease severity index (DSI) from 6 to 16 plants per accession (S1 File). Observed phenotypes covered most of the range (Fig 1A). The most resistant accession was Ei2 (6915) with a DSI of 1.99 ± 0.39, while the most susceptible accession was ALL15 (4) with a DSI of 5.92 ± 0.14. There was no obvious structure in the geographic distribution of this phenotype (Fig 1B).

Next, we performed a genome wide association analysis using a mixed model algorithm on single nucleotide polymorphism data from the *A*. *thaliana* 250K chip (S2 File, S1 Table). This revealed two significant loci at the false-discovery rate (FDR) level of q < 1.0.e^-07^ which corresponds to–log_10_(p-value) of 5.25. The highest association value, -log_10_(p-value) = 6.03, corresponded to a missense SNP located on chromosome 1 (position 7 061 677) within the predicted coding sequence of the gene *At1g20380* (Fig 1C and 1D). *At1g20380* encodes an uncharacterized prolyl-oligopeptidase hereby named *POQR* (Prolyl-Oligopeptidase associated with Quantitative Resistance).

### The *poqr-1* and *poqr-2* mutant lines are impaired in quantitative disease resistance against *S*. *sclerotiorum*

To directly test the role of *POQR* in quantitative disease resistance against *S*. *sclerotiorum*, we analyzed the phenotype of two Col-0 insertion mutant lines. The *poqr-1* line (SALK_121407C) and the *poqr-2* line (SALK_027815C) had a T-DNA insertion in the second and tenth exon of the *POQR* gene respectively (S1 Fig). We detected truncated transcripts in healthy plants of both lines, encoding proteins truncated after amino acids 53 (*poqr-1*) and 681 (*poqr-2*), instead of 732 in the wild type protein. Quantitative RT-PCR analysis showed that *POQR* was induced ~5.2 times upon *S*. *sclerotiorum* infection in Col-0 wild type plants but this induction was abolished in *poqr* mutants (S1 Fig). The *poqr-1* and *poqr-2* mutant lines showed no obvious macroscopic developmental and growth defects (S1 Fig).

**Fig 2:**
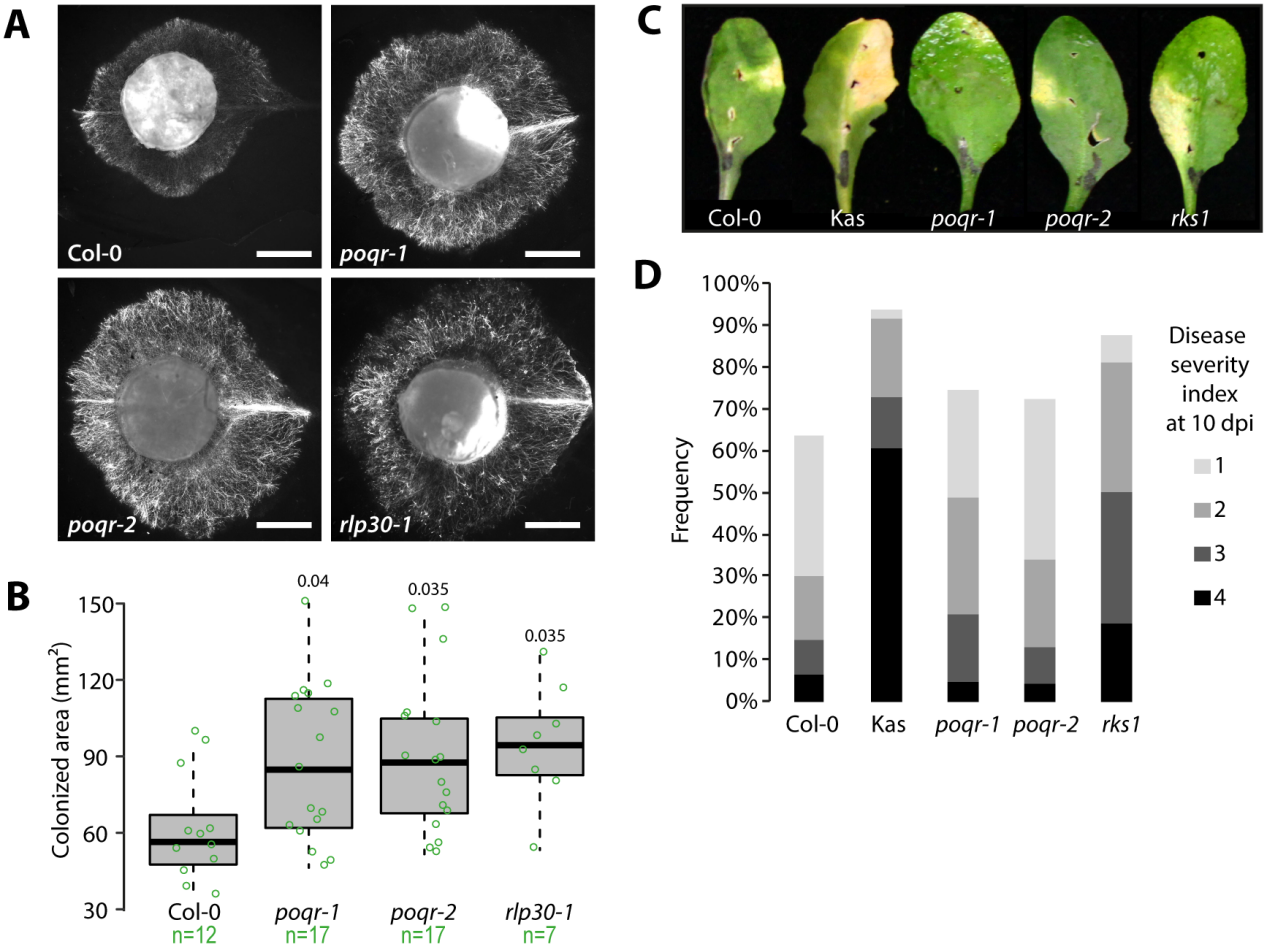
POQR confers enhanced quantitative disease resistance against *S*. *sclerotiorum*. **(A)** Representative pictures of area colonized by *S*. *sclerotiorum* expressing GFP, 24 hours after inoculation. Bar = 2.5 mm. **(B)** Measure of leaf area colonized by *S*. *sclerotiorum* 24 hours after inoculation. Values are shown for n = 7 to 17 individual plants per genotype from two independent biological experiments. Significance of the difference from Col-0 was assessed by a Student’s t test with Benjamini-Hochberg correction for multiple testing (p-values indicated above boxes). **(C)** Pictures of representative symptoms on *A*. *thaliana* leaves 10 days after inoculation by *X*. *campestris* pv. *campestris*. **(D)** Proportion of plants presenting a disease severity index of 1, 2, 3 or 4 at 10 days after inoculation (dpi) by *X*. *campestris* pv. *campestris*. Counts from n = 32 to 48 individual plants per genotype from three independent biological experiments.

We used a strain of *S*. *sclerotiorum* constitutively expressing the green fluorescent protein to determine the extent to which *POQR* affects the ability of *S*. *sclerotiorum* to colonize *A*. *thaliana* leaves (Fig 2A and 2B). The average area colonized by the fungus 24 hours after inoculation was ~61 mm^2^ in the Col-0 wild type, ~96 mm^2^ in the susceptible mutant *rlp30-1* [19], ~87 mm^2^ and ~90 mm^2^ in the *poqr-1* and *poqr-2* mutants (Student’s t test with Benjamini-Hochberg correction p-value = 0.04 and 0.035 respectively). Therefore, *POQR* disruption results in a clear increase in leaf colonization by *S*. *sclerotiorum*. In agreement, we measured an increase in disease lesion size on *poqr* mutant lines compared to wild type 36 hours after inoculation by *S*. *sclerotiorum* isolate 1980 (S1 Fig). In these assays, lesions on *poqr1* were intermediate between Col-0 and *poqr2*. Consistent with previous report on natural accessions susceptibility to *S*. *sclerotiorum* [17], Rubenzhnoe showed the smallest and Shadahra the largest lesions in our experiments.

To test whether *poqr* mutants are altered in a general biotic stress response pathway, we challenged *A*. *thaliana* wild type, control and *poqr* mutant plants with the bacterial pathogen *Xanthomonas campestris* pv. *campestris* (Fig 2C and 2D). We scored symptoms following the DSI method of [21] at 10 days post inoculation. Severely diseased plants (DSI 3 or 4) were limited to 15% of Col-0 wild type plants at this time. As expected, the Kashmir accession and *rks1-1* mutant appeared clearly more susceptible than the wild type, with 73% and 50% of plants showing a DSI 3 or 4 respectively [21]. Severely diseased plants (DSI 3 or 4) represented 21% of *poqr-1* plants, and 13% of *poqr-2* plants, similar to the wild type. To broaden our view of the plant pathogens to which POQR respond, we exploited publicly available *A*. *thaliana* gene expression data (S2 Fig). These data suggested that *POQR* is induced rather specifically during the response to leaf-infecting necrotrophic fungal pathogens such as *Botrytis cinerea* and *Alternaria brassicicola*. These results confirm the identification of a new genetic component of *A*. *thaliana* QDR and demonstrate a positive and relatively specific role for *POQR* in QDR against *S*. *sclerotiorum*.

### *POQR* sequence polymorphisms associate with QDR in *A*. *thaliana*

**Fig 3:**
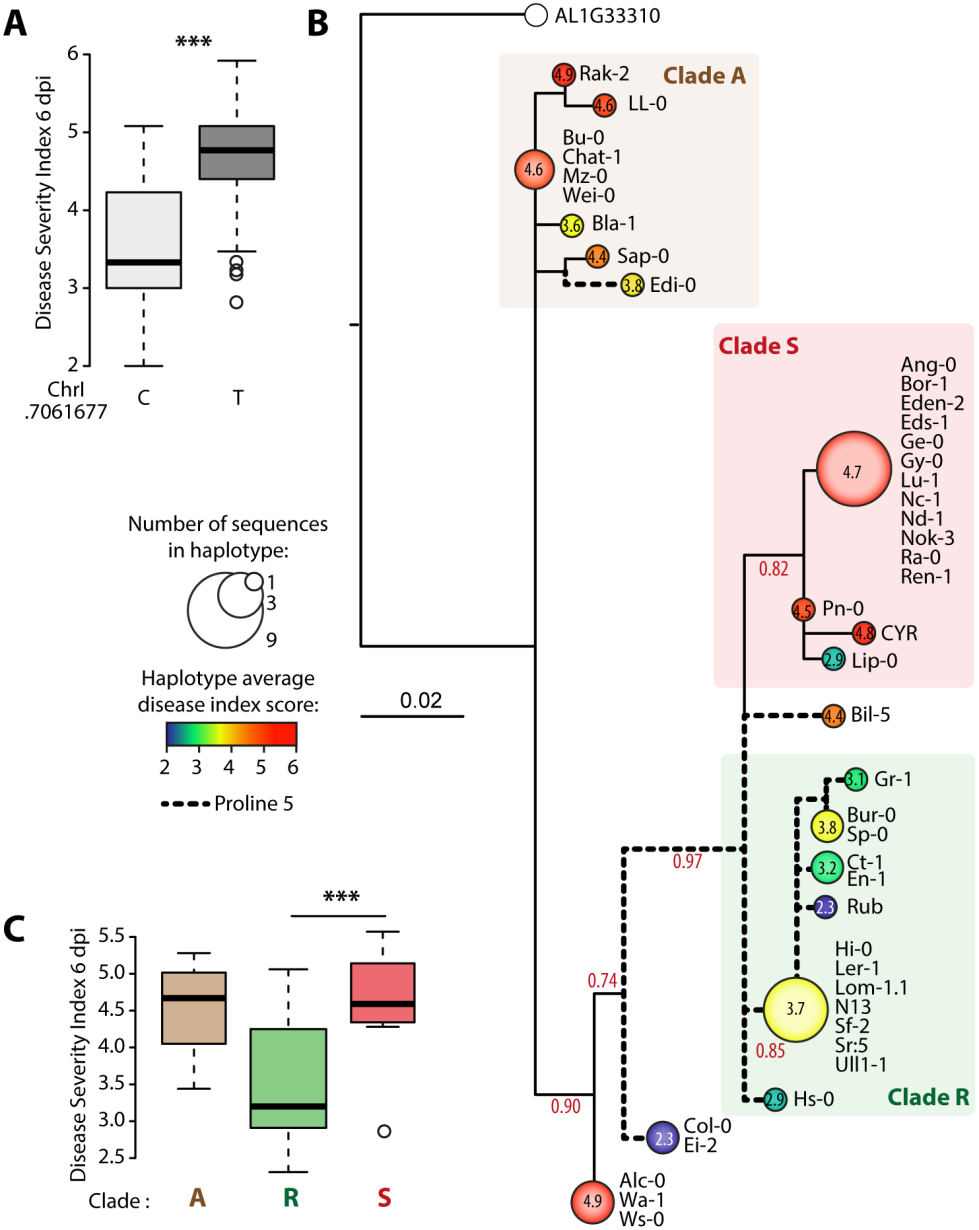
Natural diversity at *POQR* locus in *A*. *thaliana* associates sequence and expression polymorphism with QDR. (A) Distribution of disease severity index for accessions harboring either a C or a T at position 7061677 of chromosome 1, corresponding respectively to a proline or serine at POQR amino acid position 5 (*** Student’s t test p-value<0.01). **(B)** Maximum likelihood phylogenetic tree of POQR protein sequences in 46 *A*. *thaliana* accessions. Identical sequences were collapsed; nodes are sized proportionally to the number of accessions with identical POQR isoform, and colored according to the average disease severity index after *S*. *sclerotiorum* inoculation, indicated at the center of each node. Nodes are labeled with accessions forming the corresponding group. Portions of the network corresponding to clades A, S and R are highlighted with colored background. Branches carrying POQR isoforms with a proline at position 5 are shown as bold dotted lines. Support corresponding to an SH-like approximate likelihood-ratio test is shown for major branches. **(C)** Distribution of disease severity index 6 days after *S*. *sclerotiorum* inoculation in major POQR clades (*** Student’s t test p-value<0.01).

To get insights into the link between POQR natural diversity and its function in QDR, we first analyzed the distribution of DSI in *A*. *thaliana* accessions harboring either a cytosine or a thymine at the top associated SNP in our GWA mapping (position 7061677 on chromosome I) (Fig 3A). The median DSI was ~3.3 for accessions with a cytosine and ~4.8 for accessions with a thymine at this position (Student’s t test p-value = 1.3e^-05^).

Next, we analyzed the natural diversity of POQR protein sequence in 46 accessions from our European GWA population [29]. We verified by PCR sequencing the N-terminal sequence of *POQR* in 8 *A*. *thaliana* accessions (S3 File). We constructed a maximum likelihood phylogenetic tree with POQR sequence from 46 European accessions plus the Col-0 accession, and used *A*. *lyrata* POQR sequence (AL1G33310) to root the tree (Fig 3B, S4 File). This revealed three major well-supported clades, including 9, 14 and 15 POQR sequences. We mapped average DSI for each POQR isoform to highlight contrasted DSI values in each clade. Clade A (‘Ancestral’) includes 9 accessions with median DSI of 4.6, clade R (‘Resistant’) includes 14 accessions with median DSI of 3.2 and clade S (‘Susceptible’) includes 15 accessions with median DSI of 4.5. The divergence of clades R and S associates with significant differences in DSI (Student’s t test p-value = 2.9e^-03^) and involved the substitution of POQR serine 5 into a proline (S5P polymorphism), which corresponds to the SNP with the highest association value in our GWA analysis (Fig 3C). The S5P polymorphism showed a discontinuous phylogenetic distribution. Accessions containing the S5P polymorphism are more resistant than sister lineages. The S5P polymorphism is notably common to all accessions in clade R, which includes 8 of the 10 most resistant accessions included in this analysis.

### Convergent sequence evolution of *POQR* homologs in multiple plant lineages

**Fig 4:**
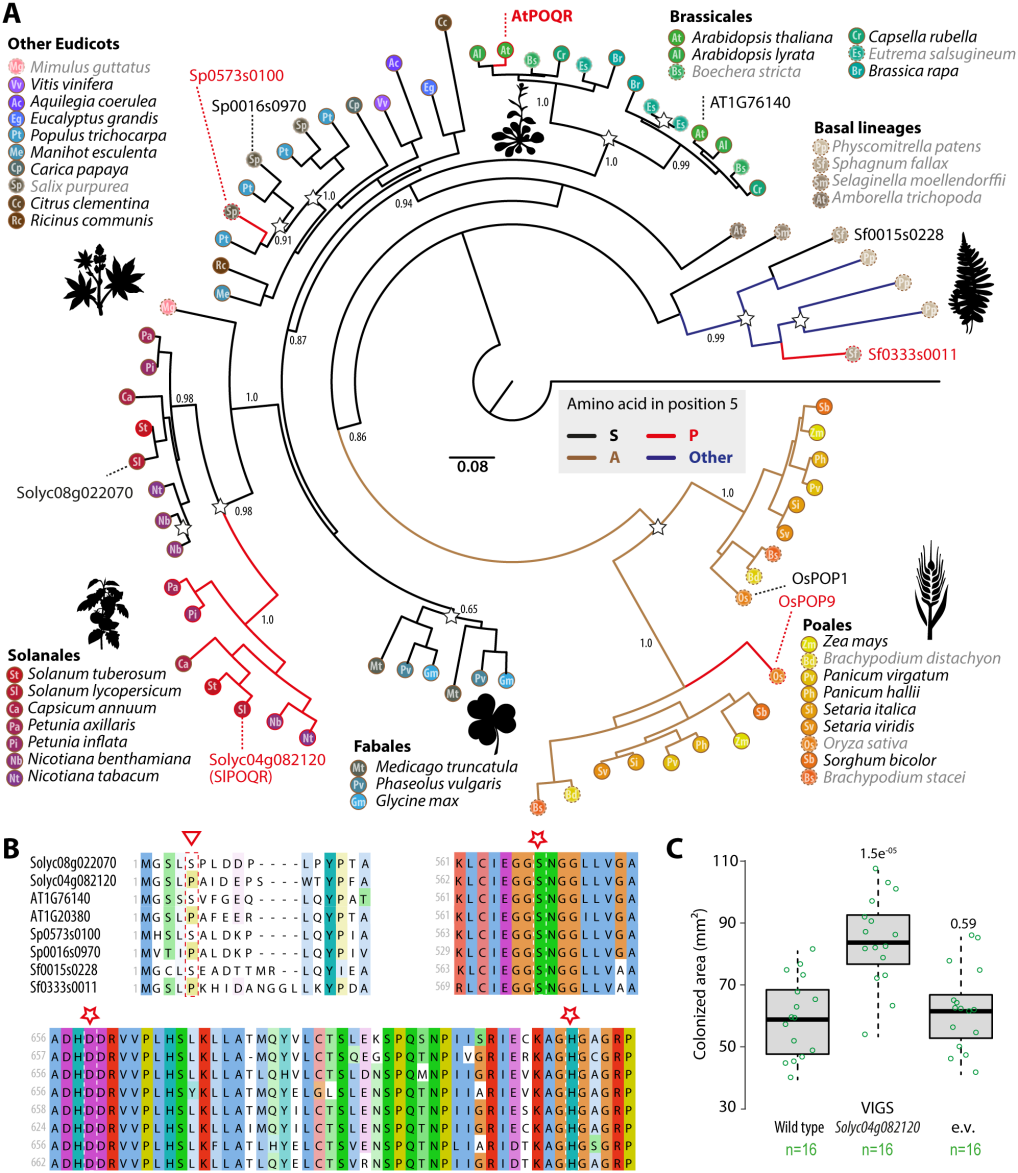
Parallel sequence evolution of POQR homologs in multiple plant lineages. **(A)** Maximum likelihood phylogenetic tree including the 75 best *POQR* homologs from 40 plant species. Terminal nodes are colored and labeled according to plant species, following the legend shown around the tree. Species belonging to a genus for which *S*. *sclerotiorum* infection has not been reported are shown with dotted nodes and labeled in grey (data from https://nt.ars-grin.gov/fungaldatabases/). Support corresponding to an SH-like approximate likelihood-ratio test is shown for major branches. Branches are colored according to amino acid in position 5 of POQR sequences. POQR homologs from *Sphagnum fallax*, *Arabidopsis thaliana*, *Solanum lycopersicum*, *Salix purpurea* and *Oryza sativa* are labeled with corresponding identifiers from the Phytozome database. **(B)** Multiple sequence alignment of POQR homologs from *Sphagnum fallax*, *Arabidopsis thaliana*, *Solanum lycopersicum*, *Salix purpurea* and *Oryza sativa* showing diversity at position 5 (triangle) and conservation of the catalytic triad (stars). **(C)** Measure of leaf area colonized by *S*. *sclerotiorum* 24 hours after inoculation in wild type tomato plants, plants silenced for POQR by virus induced gene silencing (VIGS) and plants carrying the empty viral vector (e.v.). Values are shown for n = 16 individual plants per genotype from two independent biological experiments. Significance of the difference from wild type was assessed by a Student’s t test with Benjamini-Hochberg correction for multiple testing (p-values indicated above boxes).

To explore POQR diversity across plants, we searched for *POQR* homologs in the complete genome of 40 plant species and constructed a maximum likelihood phylogenetic tree using *Volvox carteri POQR* homolog as a root (Fig 4A, S5 File). We retrieved a total of 75 *POQR* homologs, with 11 species harboring a single *POQR* homolog, 24 species harboring two *POQR* homologs, 4 species with three *POQR* homologs and one species with four *POQR* homologs (S5 File). The most parsimonious tree defined six well delimited clades corresponding to (i) Bryophyta, (ii) Brassicales, (iii) Solanales, (iv) Fabales, (v) other Eudicots and (vi) Poales, suggesting that for genomes including multiple *POQR* paralogs, duplications are posterior to the divergence of the corresponding plant orders. The Brassicales, Solanales, Fabales and Poales clades showed clear duplication patterns in all species analyzed. This indicates parallel duplication of *POQR* ancestral gene early in the evolution of Brassicales, Solanales Fabales, and Poales, consistent with paleo-polyploidization by whole genome duplications in several angiosperm lineages between ~75 and 25 million years ago (Mya) [30].

Polymorphism at position 5 of the POQR protein was associated with QDR against *S*. *sclerotiorum* in *A*. *thaliana*. We highlighted the evolution of position 5 in the evolution of POQR across plants (Fig 4A). Among Bryophyta and Dicotyledones, 77% of POQR proteins showed a Serine at position five, while in Monocotyledones, all but one POQR proteins had an Alanine at position five. With the exception of sequences from *P*. *patens* that had Glycines at position five, all 10 remaining POQR proteins had a Proline at position five. The genome of *Sphagnum fallax*, *A*. *thaliana*, *Salix purpurea* and 6 Solanales species, including *Solanum lycopersicum*, all contain *POQR* homologs with a Serine at position five and others with a Proline at position five (Fig 4B). Similarly, the genome of *Oryza sativa* includes one *POQR* homolog with an Alanine at position five (*OsPOP1*) and an ortholog with a Proline at position five (*OsPOP9*).

In *A*. *thaliana* Col-0 genome, At1g76140 is AtPOQR closest paralog (81.4% identity at the protein level) and harbors a Serine at position five, instead of a Proline in AtPOQR. In *S*. *lycopersicum* Heinz genome, Solyc08g022070 is the closest paralog of SlPOQR (Solyc04g082120, 78.4% identity at the protein level) and harbors a Serine at position five, instead of a Proline in SlPOQR. To study the evolutionary history of POQR sequence, we performed ancestral sequence reconstruction with FastML (S6 File). We compared POQR homologs from *Brassicaceae*, *Solanaceae* and *Poales* with their respective ancestral sequences prior gene duplication in these lineages. This revealed the occurrence of S5P and F613Y substitutions in parallel in AtPOQR, OsPOP9 and in all seven *Solanaceae* species analyzed (p-value of these substitutions occurring in three independent lineages is 7.5e^-09^). We noted two additional amino acid substitutions that occurred in parallel in AtPOQR and SlPOQR (V119I and A533S) that could have contributed to POQR sequence adaptation to function in QDR.

To support a role for *POQR* in QDR against *S*. *sclerotiorum* in tomato, we used a virus-induced gene silencing (VIGS) approach to silence *POQR* in tomato (Fig 4C). Eighteen days after delivery of the viral constructs, plants were inoculated with a GFP-expressing *S*. *sclerotiorum* strain. Plants carrying the *POQR*-silencing construct showed an average ~57% reduction in *POQR* expression during *S*. *sclerotiorum* infection as compared to wild type plants and plants carrying the empty viral construct (S3 Fig). The area colonized by the fungus 24 hours after inoculation was measured using fluorescence imaging. Consistent with results obtained in *A*. *thaliana poqr* mutant lines, we found that *POQR* silencing in tomato resulted in colonized areas ~1.42 times larger than in wild type plants and ~1.36 times larger than plants carrying the empty viral construct. These results indicate parallel duplication and convergent sequence evolution of *POQR* homologs in multiple plant lineages.

### Parallel evolution of *POQR* gene induction upon *S*. *sclerotiorum* infection in *A*. *thaliana* and *S*. *lycopersicum*

**Fig 5:**
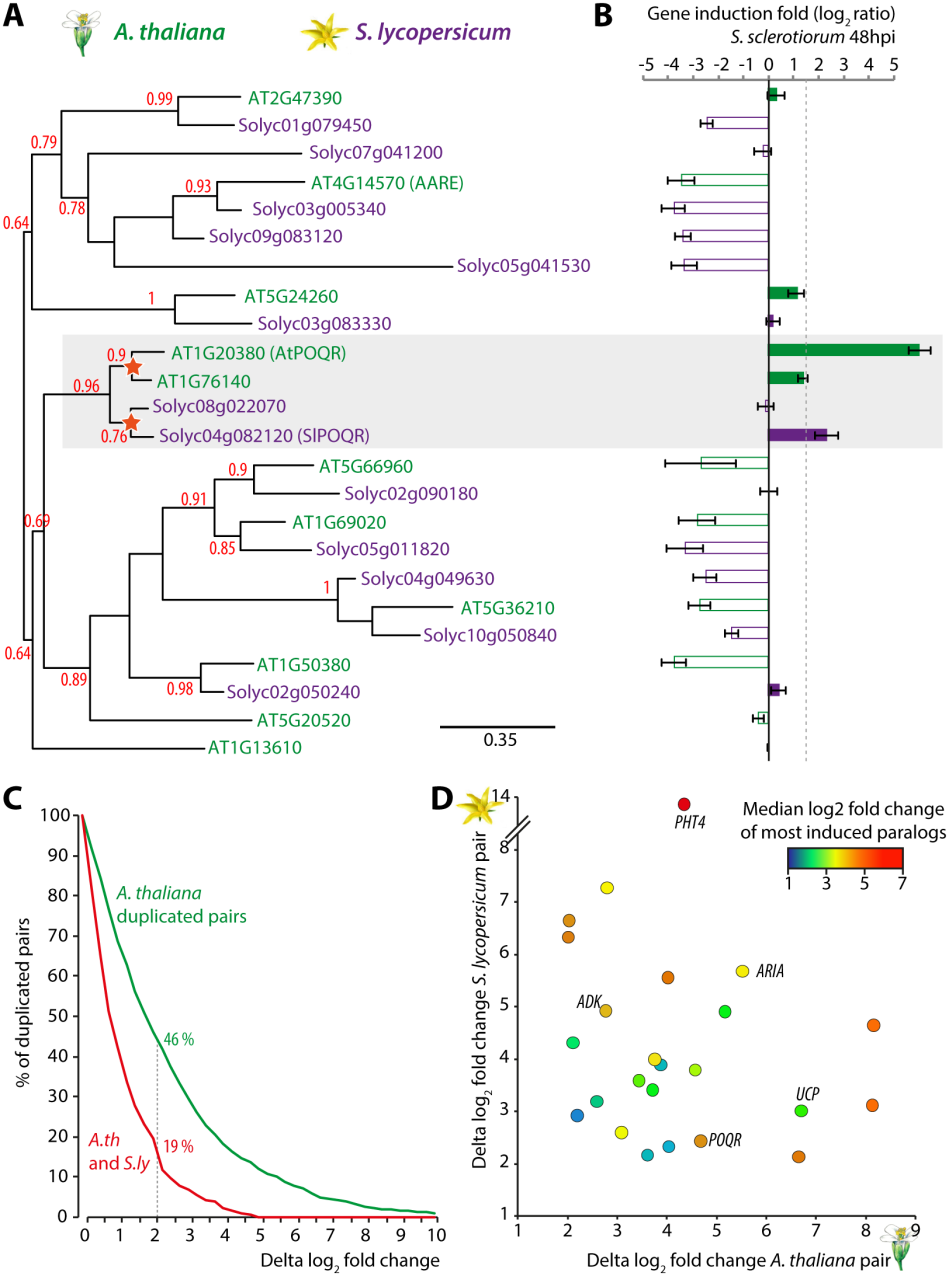
Parallel evolution of *POQR* gene induction upon *S*. *sclerotiorum* infection in *A*. *thaliana* and *S*. *lycopersicum*. **(A)** Maximum likelihood phylogenetic tree of *A*. *thaliana* (green) and *S*. *lycopersicum* (purple) prolyl-oligopeptidase (POP) family. Support corresponding to an SH-like approximate likelihood-ratio test is shown for major branches. Stars indicate gene duplication events. **(B)** Expression of *A*. *thaliana* and *S*. *lycopersicum POP* genes upon inoculation by *S*. *sclerotiorum*. Values show average log_2_ fold change (LFC) compared to mock-treated plants from three independent biological replicates, error bars show s.e.m. **(C)** Distribution of duplicated gene pairs according to their difference in LFC. Distribution is shown for duplicated gene pairs from *A*. *thaliana* (green, 1083 pairs) and genes duplicated both in *A*. *thaliana* and *S*. *lycopersicum* (red, 216 pairs of pairs). **(D)** Distribution of 21 clusters of gene duplicated both in *A*. *thaliana* and *S*. *lycopersicum* according to the delta LFC in *A*. *thaliana* gene pair (X-axis) and *S*. *lycopersicum* gene pair (Y-axis). The color code shows median of the two highest LFCs in a cluster. Labels refer to clusters commented in the text.

The *A*. *thaliana* Prolyl-olypeptidase (POP) family includes 11 members harboring a peptidase S9 catalytic domain (PF00326) (S4 Fig, S7 File). Two AtPOPs have been studied functionally: *At5g20520* encodes WAV2, a negative regulator of skewing root patterns [31], and *At4g14570* encodes an acyl-amino acid-releasing enzyme (AARE) mostly present in chloroplasts [32]. Five AtPOPs, including POQR, also harbor a Prolyl-oligopeptidase N-terminal beta-propeller domain (PF02897) which functions in limiting the access of the enzyme catalytic triad to small (<30 aa) unstructured peptides [33,34] (S4 Fig). In tomato, the POP family includes 13 members, four of which harbor the N-terminal beta-propeller domain (S4 Fig). In phylogenies including *A*. *thaliana* and *S*. *lycopersicum* POPs, At1g76140 and AtPOQR on the one hand, and Solyc08g022070 and Solyc04g082120 (SlPOQR) on the other hand, formed well resolved pairs, supporting the conclusion that they duplicated after the divergence of the two plant species (Fig 5A, S7 File).

To further support convergent evolution of *POQR* homologs in *A*. *thaliana* and *S*. *lycopersicum*, we first analyzed global gene expression in response to *S*. *sclerotiorum* in *A*. *thaliana* and *S*. *lycopersicum* (S8 File). We detected 4,703 genes (16.4%) significantly upregulated (log fold change LFC>1.5, adjusted p-value<0.01) and 5,813 genes (20.3%) significantly down-regulated in *A*. *thaliana*. In *S*. *lycopersicum*, 3,513 genes (10%) were upregulated and 4,556 (12.9%) were down-regulated. We analyzed gene annotations from both species and found 50 gene ontology (GO) terms significantly enriched among up-regulated genes including defense-related mechanisms, detoxification processes and secondary metabolism (S9 File). We found 136 GO terms significantly enriched among down-regulated genes mainly related to photosynthesis (S9 File).

We then focused on the expression of *POP* genes in response to *S*. *sclerotiorum* infection at the genome scale in *A*. *thaliana* and *S*. *lycopersicum* (Fig 5B, S8 File). In *A*. *thaliana*, four *AtPOP* genes were significantly (adjusted p-value<0.01) down-regulated upon *S*. *sclerotiorum* inoculation, while three genes were significantly up-regulated. In *S*. *lycopersicum*, six *SlPOP* genes were significantly down-regulated upon *S*. *sclerotiorum* inoculation, while *SlPOQR* was the only *POP* gene significantly up-regulated. Among a total of 24 *POP* genes in the two species, *AtPOQR* and *SlPOQR* were the only ones induced more than 3 fold (LFC>1.5) upon *S*. *sclerotiorum* inoculation. Both *AtPOQR* and *SlPOQR* were significantly more induced upon *S*. *sclerotiorum* inoculation than their duplicate (delta LFC>2). These results show that, after divergence of the two species, *POQR* ancestral gene duplicated in parallel in *A*. *thaliana* and *S*. *lycopersicum*, with one paralog (*POQR*) evolving responsiveness to *S*. *sclerotiorum* infection in each species.

To document divergence in duplicated genes expression upon *S*. *sclerotiorum* infection at the whole genome scale, we first analyzed the expression of 1843 *A*. *thaliana* duplicated gene pairs [35]. For this, we calculated LFC of gene expression in *S*. *sclerotiorum*-infected plants compared to mock-treated plants, and the difference between LFC for two genes forming a duplicated pair (delta LFC) (Fig 5C, S10 File). Median delta LFC was 1.77, corresponding to a ~3.4 fold change in gene expression responsiveness to *S*. *sclerotiorum* infection. A total of 852 duplicated gene pairs (46%) showed delta LFC≥2, supporting the view that divergence of gene expression is relatively widespread between duplicated genes in *A*. *thaliana* [35]. We next used Markov Cluster algorithm [36] on *A*. *thaliana* and *S*. *lycopersicum* predicted proteomes to identify 252 clusters of genes that duplicated in parallel in *A*. *thaliana* and *S*. *lycopersicum* (a total of 504 *A*. *thaliana* genes and 504 *S*. *lycopersicum* genes). This identified 42 gene clusters (19%) with a delta LFC≥2 was observed after fungal infection in both *A*. *thaliana* and *S*. *lycopersicum* (Fig 5C). Among those, 24 clusters included paralogs induced at least 4 fold both in *A*. *thaliana* and *S*. *lycopersicum* (Fig 5D, S11 File). The corresponding genes are notably involved in cell redox homeostasis (e.g. the uncoupling protein cluster *UCP*), in primary and secondary metabolism (e.g. the adenylate kinase cluster *ADK*), transport (e.g. the phosphate transporter 4 cluster *PHT4*), signaling (e.g. protein kinases), and response to abscisic acid (e.g. the ARM repeat protein interacting with ABF2 cluster *ARIA*). These genes are prime candidates for contributing to quantitative disease resistance mechanisms shared between plant species.

## Discussion

In this study, we identify the prolyl oligopeptidase *POQR* as a previously unknown determinant of QDR against the fungal pathogen *S*. *sclerotiorum* in *A*. *thaliana*. Our work associates *POQR* sequence and expression polymorphisms with contrasted resistance to *S*. *sclerotiorum*. We reveal the convergent evolution of POQR sequence in multiple plant species following independent gene duplication events. The induction of POQR gene expression upon *S*. *sclerotiorum* inoculation also evolved in parallel in *A*. *thaliana* and *S*. *lycopersicum*. A genome scale analysis of expression divergence upon *S*. *sclerotiorum* inoculation for *A*. *thaliana* and *S*. *lycopersicum* duplicated genes suggests that convergent gene expression polymorphism could have shaped several conserved components of the quantitative disease resistance machinery in these species.

### A prolyl oligopeptidase mediates QDR to *S*. *sclerotiorum*

Plant QDR is a complex trait governed by multiple genes of minor effect, which renders the identification of the underlying molecular bases challenging [4,5]. Whereas gene-for-gene resistance relies on receptor proteins that belong to the receptor-like kinase (RLK) or the Nod-like receptor (NLR) classes family, the limited number of QDR determinants known to date encodes a remarkably broad range of molecular functions, such as transporters [8,37,38], kinase domain-containing proteins [9,21] or enzymes of the central metabolism [39]. Gene variants conferring resistance to fungal pathogens in natural plant populations also remain poorly documented [40]. We report the role of a prolyl oligopeptidase (POP) conferring QDR to *S*. *sclerotiorum* in *A*. *thaliana*. Several classes of proteases are involved in plant defenses against fungal and bacterial pathogens [41–43] but little is known about POP functions in plants. In animals, unlike other serine proteases, POP cleaves short peptides (usually <30 amino acids) after a proline residue [33]. In human, this enzyme is associated with several neurological disorders, possibly through its action in the metabolism of neuropeptides or in the inositol phosphate signaling pathway [44]. In Basidiomycota fungi, POPs are required for the maturation of amatoxins, toxic cyclic peptides derived from ~30 amino acid propeptides [45,46]. Plants in the Caryophyllaceae family use a POP enzyme designated as peptide cyclase 1 (PCY1) to synthesize cyclic peptides from propeptide precursors [47]. Plant cyclic peptides are diverse and widespread, some exhibiting antifungal activity [48]. POQR may therefore be involved in the maturation of plant antifungal peptides. Alternatively, POQR may act directly on fungal secreted proteins promoting disease [10] to disable them. Our inoculation assays with the bacterial pathogen *Xanthomonas campestris* pv. *campestris* and a survey of publicly available *A*. *thaliana* gene expression data were in agreement with a possible activity of POQR on specific fungal processes. Future work aiming at determining the spectrum of pathogens impacted by POQR-mediated resistance should provide valuable insights into POQR molecular function.

### Impact of natural variation on POQR function

Our analyses associated the substitution of POQR serine in position 5 by a proline with enhanced QDR to *S*. *sclerotiorum* in *A*. *thaliana*. Changes at POQR N-terminus induced by the S5P substitution may affect the ability of POQR to form dimers, a property of some Archean POPs [49], the subcellular localization of POQR, or remotely affect the accessibility and function of POQR catalytic site. Additional amino acid substitutions occurred in parallel in POQR homologs from multiple species, notably the F613Y substitution that arose at least three times independently in *A*. *thaliana*, rice and *Solanaceae* species. This variant may create a novel tyrosine phosphorylation site important for POQR regulation, or as shown for the Y473F mutation in porcine POP [50], modulate the range of conditions in which the POQR enzyme is active. The analysis of *POQR* expression in two distantly-related plants species suggested that *POQR* induction was associated with resistance to *S*. *sclerotiorum*. This observation is consistent with resistance increasing with the level of POQR accumulation. Similarly, enhanced accumulation of a tomato chitinase was associated with resistance to *Alternaria solani* [51], and expression of the atypical kinase gene *RKS1* was correlated with QDR to *X*. *campestris* pv. *campestris* [21]. Besides, copy number variation and DNA methylation are polymorphisms altering the accumulation of gene products encoded by the *Rhg1* locus, which confers QDR to soybean cyst nematodes [38,52]. A detailed understanding of POQR molecular function will be required to establish causal relationships between polymorphism at the *POQR* locus and QDR to *S*. *sclerotiorum*. Our current data points towards *POQR* transcriptional regulation and the modulation of POQR enzymatic activity as determinants of QDR.

### Evolutionary history of a QDR gene

**Fig 6:**
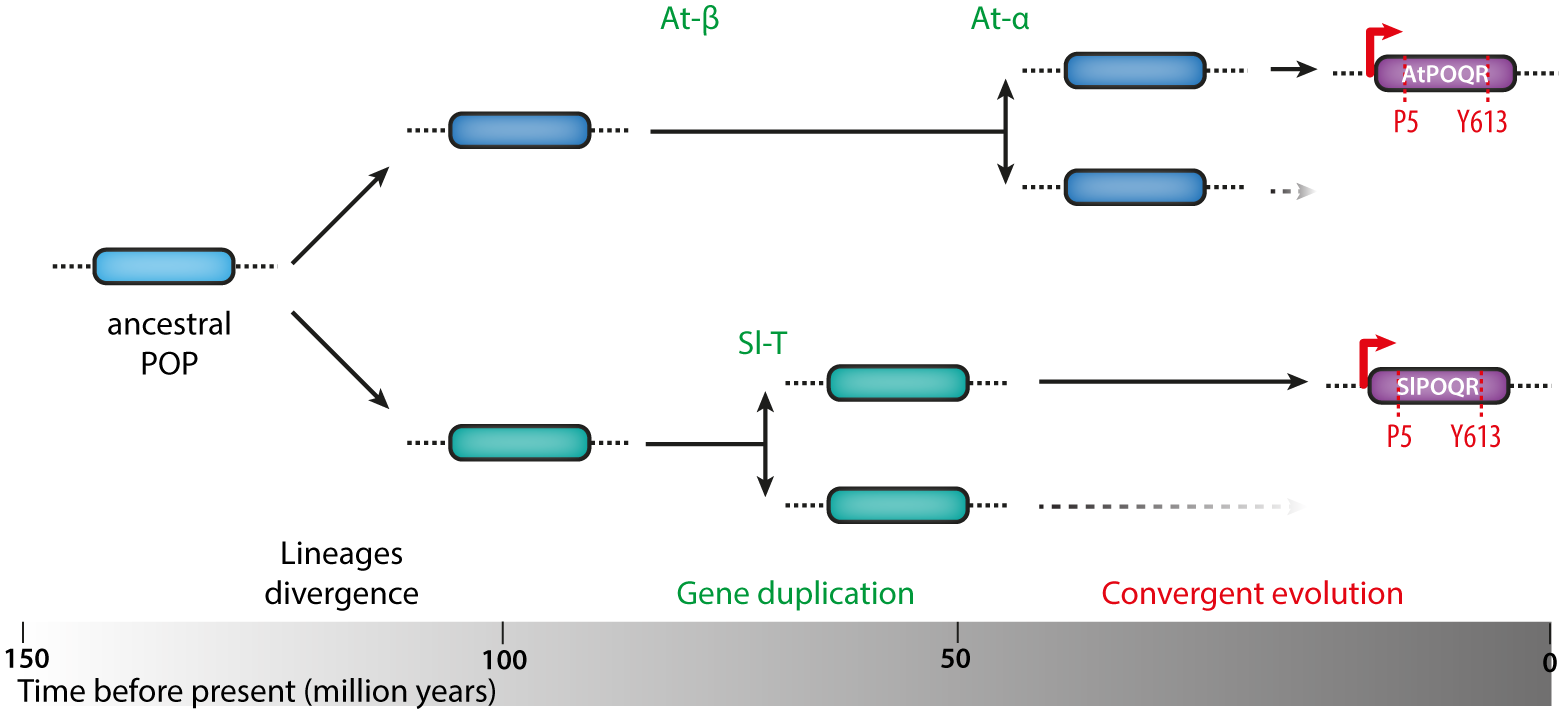
A model for convergent evolution of the *POQR* gene in *A*. *thaliana* and *S*. *lycopersicum*. Our analyses suggest that a single *POQR* ancestral gene was inherited by *A*. *thaliana* and *S*. *lycopersicum* ancestors when lineages diverged, about 120 million years ago (Mya). The ancestral POQR gene then duplicated in parallel in *A*. *thaliana* lineage between 88 and 44 Mya (At-β and At-α events) and *S*. *lycopersicum* lineage about 64 Mya (Sl-T event). After duplication, *POQR* ancestral genes underwent parallel amino acid substitutions, notably leading to the emergence of proline 5 (P5) and tyrosine 613 (Y613), and parallel gain or gene induction upon *S*. *sclerotiorum* infection (red arrow) in *A*. *thalina* and *S*. *lycopersicum* lineages.

Ancient whole genome duplication (WGD) events have had a major role in shaping the gene content of extant plant genomes, and contributed to the evolution of a range of new gene functions [53,54]. Based on our analyses, a scenario for the convergent evolution of *POQR* and its recruitment in plant QDR can be inferred (Fig 6). The Brassicales and Solanales lineages probably inherited a single *POQR* ancestral sequence at the time these two lineages diverged, about 120 million years ago (Mya) [22,55]. Two WGD events were detected in the Brassicales (At-α and At-β) estimated respectively to ~40 and ~88 Mya [54,56]. A WGD occurred in the Solanales (Sl-T) and is estimated at least ~64 Mya [55,57]. The duplication of *POQR* ancestor in *A*. *thaliana* and *S*. *lycopersicum* lineages therefore occurred at least 40 million years ago. The gene content of extant plant genomes is the result of frequent loss of duplicates created by WGDs [53]. The maintenance rate of At-α duplicates has been estimated to ~14% in average, with only ~6.5% of defense-related gene duplicates being maintained [35]. The maintenance of *POQR* duplicates over >40 million years in multiple plant species supports an important role for *POQR* in plants fitness. This is in agreement with POQR locus explaining ~20% of phenotypic variation upon *S*. *sclerotiorum* infection in the *A*. *thaliana* population analyzed in this work. After duplication, POQR ancestral genes underwent a number of parallel amino acid substitutions in the *Brassicales* and the *Solanales*, including the S5P and F613Y substitutions (Fig 6). These substitutions are present in all *POQR* homologs from Solanales, and therefore likely occurred in the most recent common ancestor, at least ~30 million years ago (estimated divergence time for *Petunia* genus, [58]). By contrast, in the Brassicales, *POQR* S5P and F613Y substitutions were only found in a subset of *A*. *thaliana* accessions, placing their probable emergence after *A*. *thaliana* divergence ~6 million years ago [59]. Remarkably, *POQR* alleles with amino acids S5 and F613 persisted in a significant number of *A*. *thaliana* accessions. The co-selection of two gene variants within a population, such as observed for *POQR*, often results from complex evolutionary constrains referred to as balancing selection [28].

### Forces driving *POQR* parallel evolution in plants

Our results suggest that plants adapted to high fungal disease pressure by tuning POQR enzymatic activity and evolving gene induction upon fungal infection. We observed similar molecular mechanisms for *POQR* evolution in *A*. *thaliana* at the infra-specific level and following duplication in the Solanales ancestor, between 30 and 64 million years ago. The estimated emergence of POQR S5P and F613Y polymorphisms in Solanales predates the inclusion of these species in *S*. *sclerotiorum* host range [13], suggesting that POQR evolution in this lineage was driven by the interaction with other fungal pathogens, or other environmental constraints. Pigmentation in mammals is a well known example of phenotypic convergence at different levels, including convergence at the level of mutation (mutational convergence) [60]. Indeed, the function of the *Mc1r* gene product was evolved similarly in beach mouse and woolly mammoth through the same R65C mutation [61,62]. In insects, the ability to feed on plants producing cardenolide toxic compounds results from parallel amino acid substitutions in the ATPα1 subunit of a Na^+^ and K^+^ transporter, independent duplications and convergent expression polymorphism of the corresponding gene [63]. Similarly our work associate parallel mutations, gene duplication and convergent expression polymorphism in *POQR*.

Epistasis is thought to reduce the rate of molecular convergence with species divergence [64]. Nevertheless, mutational convergence at the intra- and inter-specific level has been documented recently for color vision in stickleback fishes, as an adaptation to blackwater [65]. Our study provides another example of mutational convergence at the intra- and inter-specific level. The co-existence of divergent alleles within plant populations has often been associated with fitness trade-offs, notably between growth and defense [66]. Quantitative disease resistance has been proposed to rely in part on genes controlling plant growth in the absence of pathogens [4]. Such a function seems unlikely for *POQR*, considering that *A*. *thaliana poqr* mutant lines did not show obvious developmental defects. Instead, *POQR* may have specialized to function in QDR through sequence and expression polymorphisms while its duplicate copy maintained function in the control of plant growth. Partial functional redundancy between POQR and its duplicate could explain the persistence of multiple variants in *A*. *thaliana* populations and across plant species [67]. Such pleiotropic constraints have been proposed to facilitate convergent and parallel molecular evolution [64]. In agreement, we found that 19% of genes duplicated both in *A*. *thaliana* and *S*. *lycopersicum* evolved responsiveness to *S*. *sclerotiorum* in a convergent manner. Insights into the possible trade-offs mediated by these genes will be required to test for the interaction between pleiotropy and molecular convergence.

In summary, our work has shown that the evolution of genes mediating plant quantitative disease resistance displays some level of repeatability and predictability. This finding has implication for our understanding of the evolution of quantitative traits in plants and for the design of innovative and sustainable crop breeding strategies. Adaptive evolution of *POQR* involved a combination of gene copy number variation, sequence and expression polymorphism. An important question for the future will be to assess the impact of convergent mutations on POQR function in several plant species and test for the role of putative fitness trade-offs in constraining the evolution of QDR genes.

## Materials and methods

### Plants cultivation and molecular characterization

*Arabidopsis thaliana* accessions and mutant plants were obtained from the European Arabidopsis Stock Centre (NASC), ecotype ids for natural accessions used in this study are listed in S1 File. All *Arabidopsis* mutant lines used in this study were in the Col-0 background, the Nottingham Arabidopsis Stock Centre accession number N1093 Col-0 was used as wild type. Plants were grown in Jiffy pots under controlled conditions at 22°C, with a 9 hour light period and a light intensity of 120 μmol/m^2^/s 4 weeks prior to infection. Homozygous T-DNA insertion mutant plants *poqr-1* (SALK_121407C) and *poqr-2* (SALK_027815C) were isolated by PCR screening with the primers FWD_poqr1 (5’-ATGTTGGTTGAGTTGACGGAG-3’) and REV_poqr1 (5’-TTGATCAGTCCCAAGGAAATG-3’) or FWD_poqr2 (5’-GATCTCTTTGGGTGTGCTCTG-3’) and REV_poqr2 (5’- CTTGTATCGATGAGCTGGCTC-3’). The accumulation of POQR transcript in *poqr-1* and *poqr-2* plants was investigated by quantitative RT-PCR with primers F_Cpoqr (5’-GTATCGAAGGTGGTAGTAACG-3’) and R_Cpoqr (5’-TCAGAAGTCCAAGCATGTCC-3’). RNA extraction, reverse transcription and cDNA quantification were performed as described in [17], using the *At2g28390* gene as housekeeping gene [68]. Samples from three different plants per line were harvested and analyzed separately.

### Pathogens inoculations and disease scoring

For genome wide association mapping, inoculations and disease scoring were performed as in [17] on 6 to 16 plants per accessions in a randomized complete block design. For mutants phenotyping, the *S*. *sclerotiorum* strain 1980 was grown on minimal medium plates with 50mM glucose as carbon source. An agar plug (5mm in diameter) containing actively growing *S*. *sclerotiorum* mycelium was placed on the adaxial surface of leaves and plants were maintained at 80% humidity in Percival AR-41L3 chambers under the same day/light condition as for plant growth, in trays closed with parafilm to control for humidity. Pictures were taken at ~24 hours after inoculation before lesions reached leaves border on the most susceptible plants. Images were analyzed with the ImageJ software to determine lesion sizes. To assess fungal colonization, plants were inoculated by the *S*. *sclerotiorum* strain 1980 expressing GFP fused to the endogenous *OAH1* gene as described above. Pictures were taken with a Zeiss Axio Zoom V16 microscope under bright light and fluorescent illumination, and areas measured with ImageJ. Fully developed lesions that have not reached the leaves borders were analysed. *Xanthomonas campestris* inoculation assays were performed as described in [21].

### Genome wide association mapping

We performed genome-wide association mapping using the SNP database from Atwell et al. [20], which documents over 214,000 SNPs (an average of 1 SNP/500 bp) in 84 different inbred lines from the wild. We filtered the database to include only SNPs with a minor allele frequency greater than 0.10, which left 158,054 SNPs for mapping. The analysis was conducted using the Accelerated Mixed Model (AMM) approach in GWAPP [69]. To account for multiple simultaneous tests we calculated genome-wide FDR (q-values). For this, observed phenotype values were randomly assigned to *Arabidopsis* accession in 100 bootstrap replicates and p-values calculated for each SNP using the AMM approach for each bootstrap dataset, followed by Bonferroni correction.

### Natural *POQR* diversity in *A*. *thaliana* and conservation across plants

The sequence of the *POQR* gene for 42 *A*. *thaliana* accessions was retrieved from [29]. For PCR sequencing, we extracted genomic DNA from 4 week old plants of Col-0, Ge-0, Hs-0, Lip-0, Ra-0, Rak-2, Sap-0 and Wei-0 accessions as described in [21] and amplified the N-terminus of the *POQR* gene using the primers F_UTR_poqr (5’-AACGCCAACTACTTCGTCAA-3’) and RGW_poqr (5’- AGAAAGCTGGGTCCTAGTCGATCCATGAAGCATC-3’). The *A*. *lyrata* POQR homolog was retrieved from Phytozome v12 [70]. The sequences of *POQR* homologs from 40 plant species were retrieved using BlastP searches against the Phytozome v12 [70] and Solgenomics [71] databases with AtPOQR sequence as a query and with 1e^-30^ as a p-value cutoff. Multiple sequence alignments were generated with MUSCLE [72] and manually curated. The phylogenetic trees were generated in phylogeny.fr [73] by curating alignment positions with gaps, using the PhyML maximum likelihood method and an SH-like approximate likelihood-ratio test (aLRT) with the WAG substitution model for estimating branch support.

### Virus induced gene silencing

The *SlPOQR* silencing construct was designed using the SGN VIGS tool [74] and obtained by PCR amplification of a 250-bp fragment using primers Solyc04g_F_VIGS (5’- CGGGAATTCTAAAAAACTCACAAAATTGTTC-3’) and Solyc04g_R_VIGS (5’- CGGCTCGAGTCCACTTGAACTTATACCATAT-3’) containing EcoRI and XhoI restriction sites respectively. This construct was introduced into the pTRV2 vector as described in [75]. Ten days old tomato plants (*Solanum lycopersicum* L. cv Heinz) were vacuum-infiltrated with *Agrobacterium tumefaciens* GV3101::pMP90 strains carrying the pTRV2-*NbPDS*, pTRV2-*SlPOQR* or empty pTRV2 vector control mixed with strains carrying the pTRV1 vector as described in [75]. Plants were inoculated by the *S*. *sclerotiorum* strain 1980 expressing GFP 14 days after *A*. *tumefaciens* infiltration, and inoculated leaflets were imaged 24 hours after inoculation. Total RNAs from distal leaflet were harvested at the time of image acquisition to verify gene silencing. RNA extraction and quantitative RT-PCR were performed as described in [76] using *SlPOQR*-specific primers qSolyc04F_1 (5’- TTCAAGTGGAAGTGATTGGGT-3’) and qSolyc04R_1 (5’- TGTCACGAGTCCAGCTAACA-3’) and Solyc08g022070-specific primers qSolyc08F_3 (5’- ACTGTTGTCCCTGGCTTTGA-3’) and qSolyc08R_3 (5’- TGTCCTTTCCAGCCACAATGA-3’). The *Solyc12g010060* gene (Translation elongation factor 5A) [77] was used as housekeeping gene.

### Phylogeny of *A*. *thaliana* and *S*. *lycopersicum* POPs

We retrieved 16 *A*. *thaliana* proteins harboring a Peptidase S9A (IPR002470) domain from Interpro 62.0 [78] among which 12 included a PFAM peptidase S9 catalytic domain (PF00326). These 12 proteins were used in a BlastP search against TAIR10 with an e-value cutoff of 10^−01^. The resulting hits were checked against the PFAM 31.0 using gathering threshold to verify the presence of a peptidase S9 catalytic domain (PF00326), identifying 11 *AtPOP* loci. We retrieved 13 *S*. *lycopersicum* proteins harboring a PF00326 domain from the ITAG3.0 annotation of the tomato genome sequence version 3 [55]. These proteins were checked against the PFAM 31.0 using gathering threshold to verify the presence of a PF00326 domain and identify additional domains, validating 13 *SlPOP* proteins. The phylogenetic trees were based on the peptidase S9 catalytic domain (PF00326) sequence as identified by PFAM 31.0. Sequences were aligned with MUSCLE [72]. The phylogenetic trees were generated as described above.

### Gene expression analyses

For quantitative RT-PCR in *A*. *thaliana* natural accessions, the experiments were performed as described above using the F_Npoqr (5’-TGCTATCGCAAACGACGAGA-3’) and R_Npoqr (5’-TCAGTCCAACTGCTCGGTTC-3’) primers. For RNA sequencing, mature leaves of 4 week-old *Arabidopsis thaliana* and *Solanum lycopersicum* (Heinz genotype) were inoculated or not with the *S*. *sclerotiorum* strain 1980 in three independent biological replicates. The edge of developed necrotic lesions (15 to 30 mm diameter) was harvested and stored in liquid nitrogen. Samples were ground with metal beads (2.5 mm) in a Retschmill apparatus (24hertz for 2x1min) before solubilizing in 1mL Trizol reagent (ThermoFisher) and left for 5 min at RT. Chloroform (200 μL) was added and mixed thoroughly before incubating for 3 min at RT. After centrifugation at ~12,000g (4°C) for 15min, the upper aqueous phase was recovered and nucleic acids were precipitated by adding 2μL GlycoBlue (Ambion) and 500μL isopropanol (10 min at -20°C). After centrifugation at ~15,000g (4°C) for 15min, pellets were washed twice with 70% ethanol before drying and resuspended in RNAse-free water. To eliminate chloroform traces, water resuspended nucleic acids were further cleaned using an RNA extraction kit (Quiagen) following manufacturer’s instructions. Genomic DNA was removed by DNase treatment (TURBO DNase; Ambion) following manufacturer’s instructions. The quality and concentrations of RNAs preparations were assessed with an Agilent apparatus and chips (nano). Messenger RNAs sequencing was outsourced to Fasteris SA (Switzerland) to produce Illumina reads (1 x 125bp) using a HiSeq 2500 instrument (TrueSeq libraries). Reads were mapped on the TAIR10 version of *A*. *thaliana* genome or version 3 of *S*. *lycopersicum* genome using the RNA-Seq Analysis function of the CLC Genomics software with default settings. All raw and normalized RNA-seq data has been deposited in GEO (GSE106811). Total reads counts per gene were extracted and differential gene expression (log2 fold change) versus mock treated samples was calculated using the Bioconductor/DESeq2 (version 1.8.2) package. Log fold changes were quantile normalized using the preprocessCore package in R. We used the average of three biological replicates as raw gene expression values. GO enrichment analysis was performed using Fisher’s exact test implemented in the Blast2GO software [79], with annotations from Ensembl plants and using a using a 0.01 false discovery rate (FDR) threshold.

### Duplicated genes analyses

The list of old and recent duplicated gene pairs in *A*. *thaliana* was retrieved from [35]. To identify genes that duplicated in parallel in *A*. *thaliana* and *S*. *lycopersicum*, we used the Markov Cluster algorithm [36] implemented in Biolayout Express 3D [80] with default parameters, minimum cluster size = 4. The output of a self BlastP search with *A*. *thaliana* and *S*. *lycopersicum* complete proteomes with an e-value cutoff 1.0e^-90^ was used as input. Clusters containing two *A*. *thaliana* and two *S*. *lycopersicum* genes were considered for further analyses. Delta LFC was calculated as the difference between LFC for two duplicated genes in a given species.

## Supporting information

S1 File Related to Fig 1: Results of *S*. *sclerotiorum* disease symptom scoring for 84 *A*. *thaliana* accessions used in genome wide association mapping. The table includes the following fields: ecotypeid, ecotype identifier number from the 1001 genome project; ecotype name; DSI, disease severity index; DSI_stdev, standard deviation for DSI; count, number of plants analyzed.
(TXT)

S2 File Related to Fig 1: Results of genome wide association mapping for quantitative resistance against *S*. *sclerotiorum* in a European population of *A*. *thaliana*. The coma-separated text file includes the following fields: chromosome, position, score, maf (minimum allele frequency), mac (minimum allele count), beta0, beta1, correlation, genotype_var_perc.
(TXT)

S3 File Related to Fig 3: N-terminal sequence of the POQR locus in 8 *A*. *thaliana* accessions obtained by sequencing PCR products in FASTA format. (TXT)

S4 File Related to Fig 3: cDNA sequences of *POQR* alleles in 46 *A*. *thaliana* accessions and *A*. *lyrata* in FASTA format; sequence alignment used to generate the phylogenetic tree of *POQR* in 46 *A*. *thaliana* accessions in clustal format; phylogenetic tree of *POQR* in 46 *A*. *thaliana* accessions in newick format. (TXT)

S5 File Related to Fig 4: Protein sequences of POQR in 40 plant species in FASTA format; sequence alignment used to generate the phylogenetic tree of POQR in 40 plant species in clustal format; phylogenetic tree of POQR in 40 plant species in newick format. (TXT)

S6 File Related to Fig 4. Ancestral sequence reconstruction of POQR in plants using FastML. Sequences of the joint reconstruction; probabilities of the joint reconstruction; tree in ancestor format.
(TXT)

S7 File Related to Fig 5: cDNA sequences of *A*. *thaliana* and *S*. *lycopersicum* proteins from the prolyl-oligopeptidase family in FASTA format; sequence alignment used to generate the phylogenetic tree of *A*. *thaliana* and *S*. *lycopersicum* proteins from the prolyl-oligopeptidase family in FASTA format; Phylogenetic tree of *A*. *thaliana* and *S*. *lycopersicum* proteins from the prolyl-oligopeptidase family in newick format. (TXT)

S8 File Related to Fig 5: Expression upon infection by *S*. *sclerotiorum* for 28,642 *A*. *thaliana* genes and 28,801 *S*. *lycopersicum* genes. The tables include the output from DEseq2 differential expression analysis, including the log_2_ fold change of gene expression in *S*. *sclerotiorum*- vs mock-treated samples and adjusted p-values for differential expression. The QuantNormLFC column contains quantile normalized log_2_ fold changes, the “POP?” column indicates whether genes encode for prolyl-oligopeptidases (1) or not (0).
(XLSX)

S9 File Related to Fig 5: GO ID, names and categories enriched both in tomato and *Arabidopsis thaliana* among upregulated or downregulated genes. Data obtained from Blast2GO enrichment analysis Fisher’s exact test with a FDR < = 0.01. Reference annotation used are *Solanum lycopersicum* (SL2.50) and *Arabidopsis thaliana* (TAIR10) from Ensembl plants.
(XLSX)

S10 File Related to Fig 5: Duplicated gene pairs from *A*. *thaliana* and *S*. *lycopersicum* analyzed in this work. The table includes 2048 *A*. *thaliana* gene pairs identified by Blanc et al. 2004, 1843 pairs had expression values for both genes; and 266 clusters of genes that duplicated in parallel in *A*. *thaliana* and *S*. *lycopersicum* identified by Markov Chain clustering in this work, 252 clusters had expression values for all four genes.
(TXT)

S11 File Related to Fig 5: Table of duplicated gene pairs with one or more genes induced at least 4 times upon *S*. *sclerotiorum* infection in *A*. *thaliana* and *S*. *lycopersicum*, and showing convergent changes in gene expression. (XLSX)

S1 Table Related to Fig 1: Top 10 SNP showing the best association score in our GWA analysis. The table provides position of the SNP, GWA score, maximum allele frequency (maf), maximum allele count (mac), predicted SNP effect, genetic context.
(XLSX)

S1 Fig Related to Fig 2: Characterization of *poqr* mutant lines. (A) POQR gene intron/exon structure, showing the position of transfer-DNA insertions in *poqr-1* and *poqr-2* mutant lines and the position of oligonucleotide primers used in this work (arrowheads). Exons are color-coded according to the protein domain they encode, as shown in B. (B) Homology model of POQR protein structure showing the different domains. (C) Relative POQR gene expression in mock- and *S*. *sclerotiorum*-treated plants determined by quantitative RT-PCR. Significant differences to expression in the corresponding Col-0 samples was assessed by a Student’s t test (** p-value<0.05). Error bars show standard deviation from three biological replicates. (D) Development phenotype of 4-weeks old *poqr* mutant plants. (E) Size of disease lesions measured 36 hours after inoculation by *S*. *sclerotiorum*. Values shown correspond to n = 12 to 21 individual plants per genotype from two independent biological experiments. Significance of the difference from Col-0 was assessed by a Student’s t test with Benjamini-Hochberg correction for multiple testing (p-values indicated above boxes).
(TIF)

S2 Fig Related to Fig 2: Expression pattern of *A*. *thaliana POQR* (At1g20380, red) and *POQR-like* (At1g76140, grey) genes in publicly available global transcriptome datasets. (A) Expression upon inoculation by the necrotrophic fungal pathogen *Botrytis cinerea* (GEO accessions GSE39598 and GSE70137). (B) Expression upon inoculation by the necrotrophic fungal pathogen *Alternaria brassicicola* and the bacterial pathogen *Pseudomonas syringae* pv. *maculicola* (GEO accessions GSE50526 and GSE45690). (C) Expression upon inoculation by the biotrophic fungal pathogen *Golovinomyces orontii* (GEO accession GSE13739). (D) Expression upon inoculation by the bacterial pathogen *P*. *synringae*, the necrotrophic fungal pathogen *A*. *brassicicola*, and the insect pathogens *Pieris rapae*, *Frankliniella occidentalis* and *Myzus persicae* (GEO accession GSE5525). (E) Expression upon inoculation by the oomycete biotrophic pathogen *Hyaloperonospora arabidopsidis* (GEO accession GSE37255). (F) Expression upon root inoculation by the necrotrophic fungal pathogen *Fusarium oxysporum* (GEO accession GSE15236). (G) Expression upon treatment by *F*. *oxysporum* protein elicitor NEP1 (GEO accession GSE4638). Values shown are log2 induction ratio compared to mock treated plants or non-inoculated plants. Error bars show standard deviation of the mean for all biological replicates available. Experiments were performed on Col-0 accession except for (E) in Ws-4. Dpi, days post inoculation; hpi, hours post inoculation.
(TIF)

S3 Fig Related to Fig 4: Molecular and phenotypic analysis of tomato plants silenced for *SlPOQR* by VIGS. (A) Phenotype of 24 days-old wild type and *A*. *tumefaciens*-infiltrated tomato plants at the time of *S*. *sclerotiorum* inoculation. Normalized relative expression of the *SlPOQR* gene (*Solyc04g082120*) (B) and its closest homolog (*Solyc08g022070*) (C) in plants scored for *S*. *sclerotiorum* colonized area (Fig 4C). Values are shown for n = 16 individual plants per treatment from two independent biological experiments. Significance of the difference from wild type was assessed by a Student’s t test with Benjamini-Hochberg correction for multiple testing (p-values indicated above boxes). (D) Representative pictures of area colonized by *S*. *sclerotiorum* expressing GFP in tomato plants 24 hours after inoculation. Bar = 2.5 mm.
(TIF)

S4 Fig Related to Fig 5: Phylogeny and domain structure of prolyl-oligopeptidases (POP) from *A*. *thaliana* and tomato. (A) Maximum likelihood phylogenetic tree of *A*. *thaliana* POPs. (B) Predicted domain structure of *A*. *thaliana* POPs. (C) Maximum likelihood phylogenetic tree of *S*. *lycopersicum* POPs. (D) Predicted domain structure of *S*. *lycopersicum* POPs.
(TIF)
