# Supplementary figures and images for: Parallel evolution of the POQR prolyl oligo peptidase gene conferring plant quantitative disease resistance
Source: PLoS Genet. 2017 Dec 22;13(12):e1007143. doi: 10.1371/journal.pgen.1007143 (PMC5757927; doi:10.1371/journal.pgen.1007143)

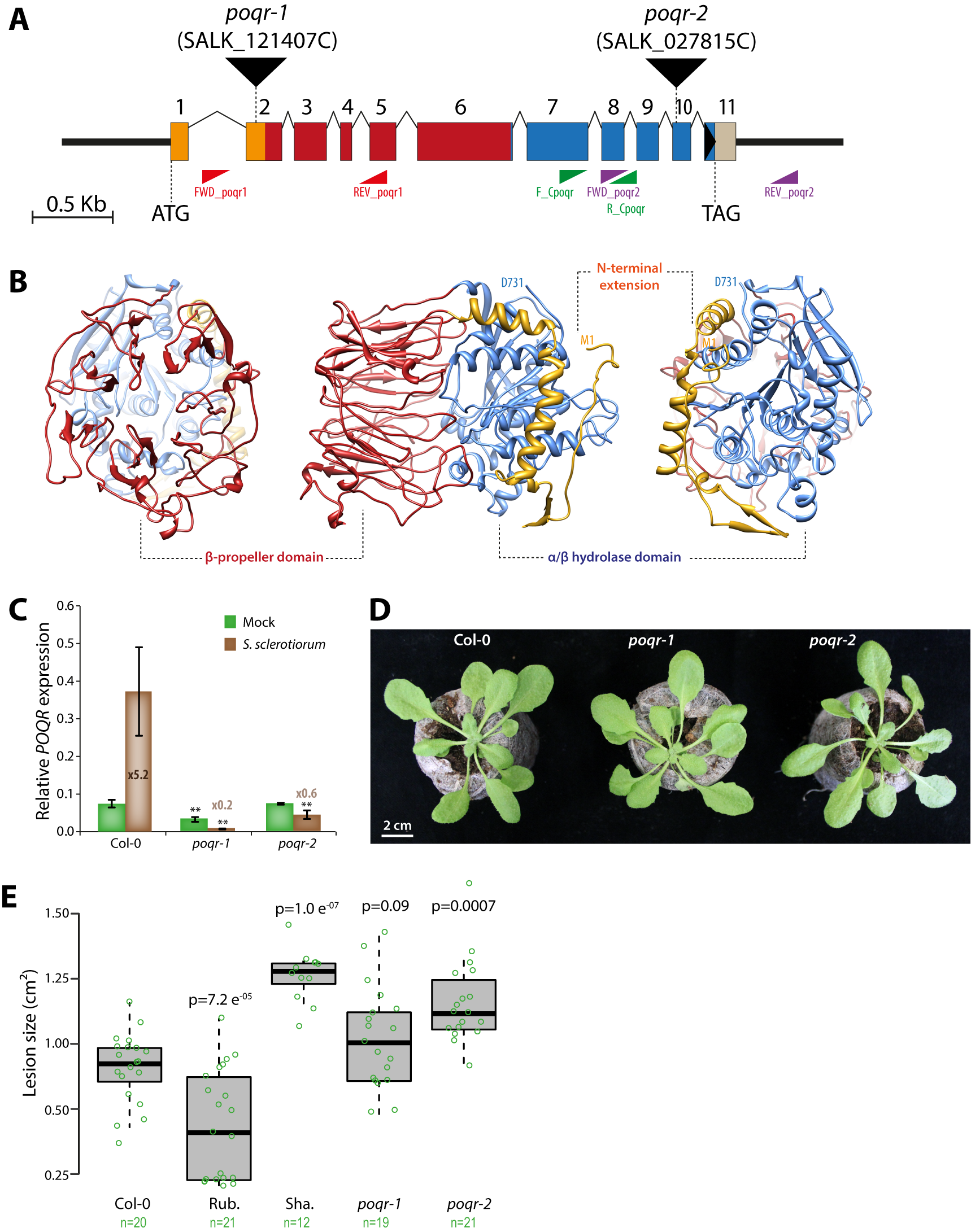

Supplement: S1 Fig — (A) POQR gene intron/exon structure, showing the position of transfer-DNA insertions in poqr-1 and poqr-2 mutant lines and the position of oligonucleotide primers used in this work (arrowheads). Exons are color-coded according to the protein domain they encode, as shown in B. (B) Homology model of POQR protein structure showing the different domains. (C) Relative POQR gene expression in mock- and S. sclerotiorum-treated plants determined by quantitative RT-PCR. Significant differences to expression in the corresponding Col-0 samples was assessed by a Student’s t test (** p-value<0.05). Error bars show standard deviation from three biological replicates. (D) Development phenotype of 4-weeks old poqr mutant plants. (E) Size of disease lesions measured 36 hours after inoculation by S. sclerotiorum. Values shown correspond to n = 12 to 21 individual plants per genotype from two independent biological experiments. Significance of the difference from Col-0 was assessed by a Student’s t test with Benjamini-Hochberg correction for multiple testing (p-values indicated above boxes). (TIF) [file pgen.1007143.s013.tif]

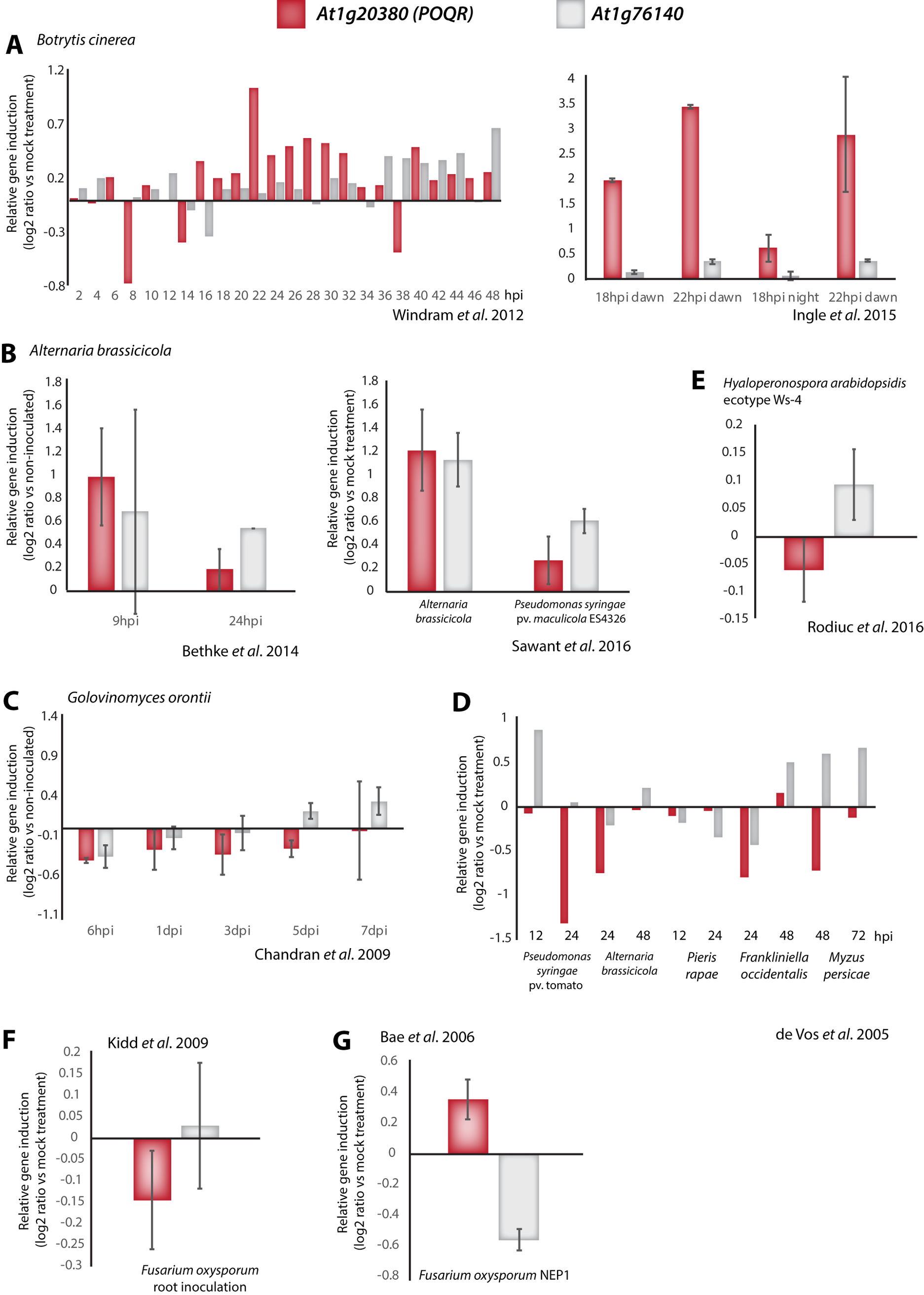

Supplement: S2 Fig — (A) Expression upon inoculation by the necrotrophic fungal pathogen Botrytis cinerea (GEO accessions GSE39598 and GSE70137). (B) Expression upon inoculation by the necrotrophic fungal pathogen Alternaria brassicicola and the bacterial pathogen Pseudomonas syringae pv. maculicola (GEO accessions GSE50526 and GSE45690). (C) Expression upon inoculation by the biotrophic fungal pathogen Golovinomyces orontii (GEO accession GSE13739). (D) Expression upon inoculation by the bacterial pathogen P. synringae, the necrotrophic fungal pathogen A. brassicicola, and the insect pathogens Pieris rapae, Frankliniella occidentalis and Myzus persicae (GEO accession GSE5525). (E) Expression upon inoculation by the oomycete biotrophic pathogen Hyaloperonospora arabidopsidis (GEO accession GSE37255). (F) Expression upon root inoculation by the necrotrophic fungal pathogen Fusarium oxysporum (GEO accession GSE15236). (G) Expression upon treatment by F. oxysporum protein elicitor NEP1 (GEO accession GSE4638). Values shown are log2 induction ratio compared to mock treated plants or non-inoculated plants. Error bars show standard deviation of the mean for all biological replicates available. Experiments were performed on Col-0 accession except for (E) in Ws-4. Dpi, days post inoculation; hpi, hours post inoculation. (TIF) [file pgen.1007143.s014.tif]

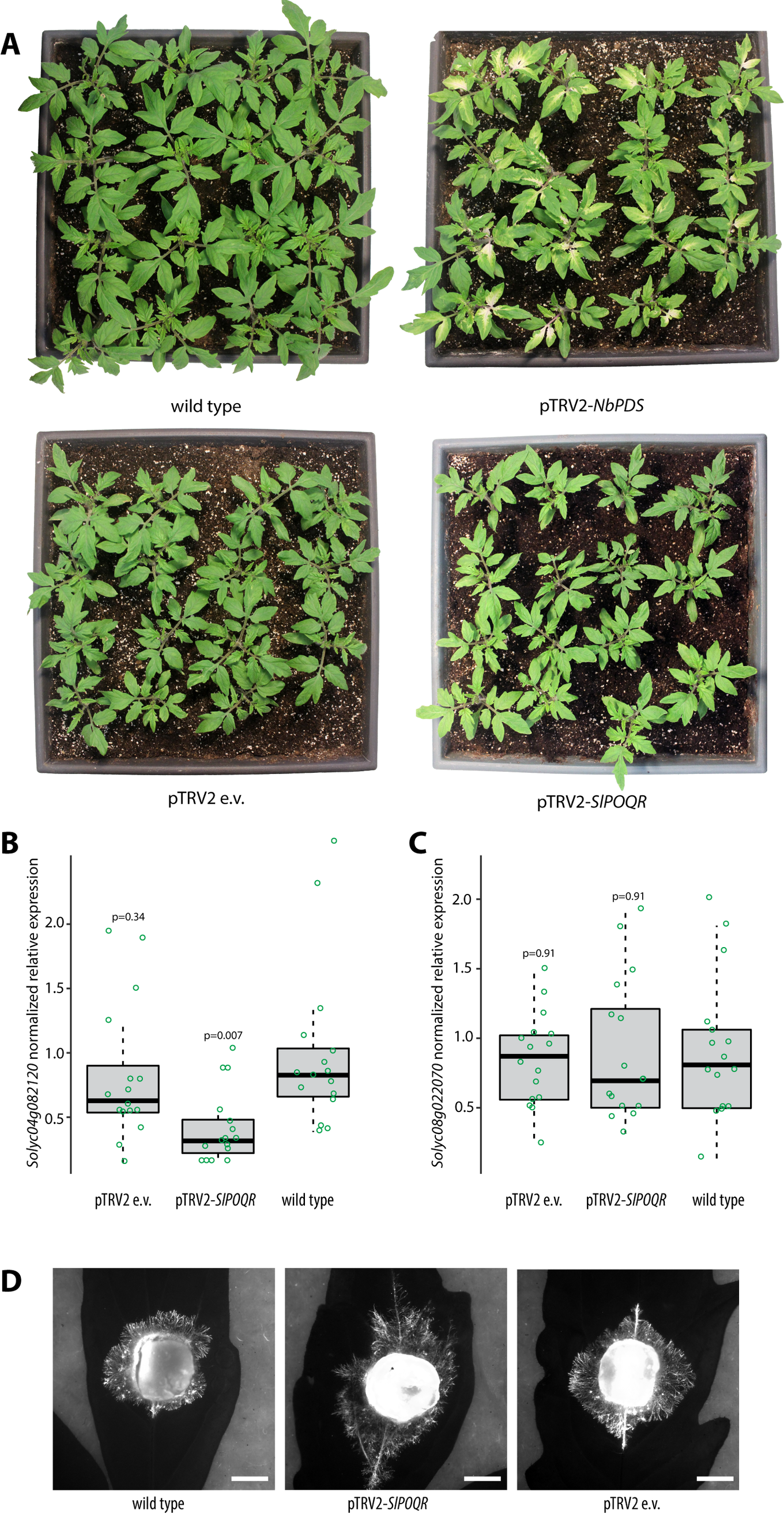

Supplement: S3 Fig — (A) Phenotype of 24 days-old wild type and A. tumefaciens-infiltrated tomato plants at the time of S. sclerotiorum inoculation. Normalized relative expression of the SlPOQR gene (Solyc04g082120) (B) and its closest homolog (Solyc08g022070) (C) in plants scored for S. sclerotiorum colonized area (Fig 4C). Values are shown for n = 16 individual plants per treatment from two independent biological experiments. Significance of the difference from wild type was assessed by a Student’s t test with Benjamini-Hochberg correction for multiple testing (p-values indicated above boxes). (D) Representative pictures of area colonized by S. sclerotiorum expressing GFP in tomato plants 24 hours after inoculation. Bar = 2.5 mm. (TIF) [file pgen.1007143.s015.tif]

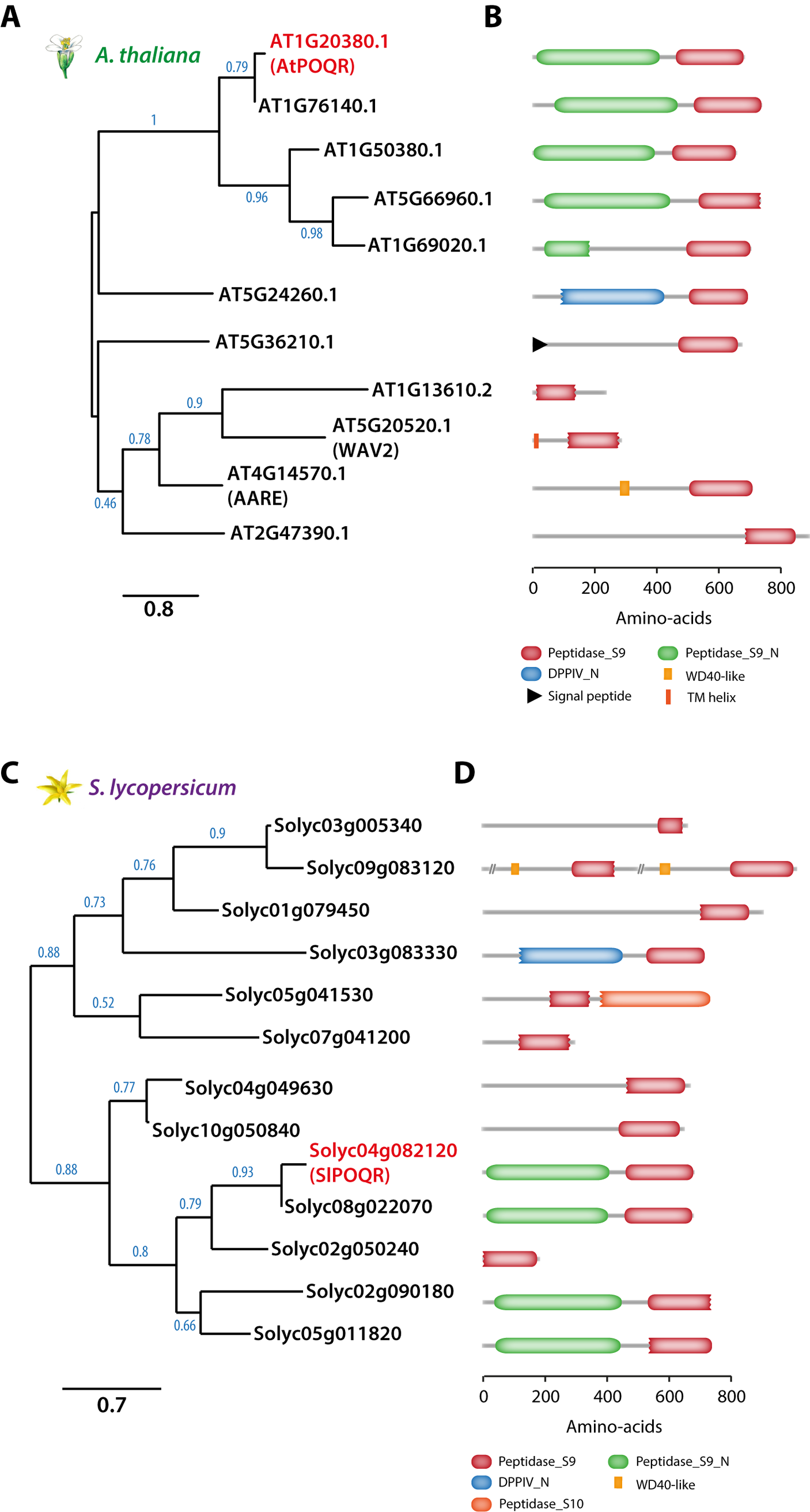

Supplement: S4 Fig — (A) Maximum likelihood phylogenetic tree of A. thaliana POPs. (B) Predicted domain structure of A. thaliana POPs. (C) Maximum likelihood phylogenetic tree of S. lycopersicum POPs. (D) Predicted domain structure of S. lycopersicum POPs. (TIF) [file pgen.1007143.s016.tif]
